# Electrocardiogram abnormalities in patients with acne undergoing isotretinoin therapy: a focused review^؆^

**DOI:** 10.1016/j.abd.2026.501304

**Published:** 2026-03-19

**Authors:** Afra Wasama Islam, Prarthana Motdhare, Shreya Mohan, Swapna Bejoy, Harsahaj Singh Wilkhoo

**Affiliations:** a Faculty of Medicine, Tbilisi State Medical University, Tbilisi, Georgia; b ClinNova international, Tbilisi, Georgia

**Keywords:** Acne vulgaris, Cardiac electrophysiology, Electrocardiography, Isotretinoin

## Abstract

Isotretinoin is an extremely successful treatment for acne, widely prescribed for moderate to severe cases unresponsive to conventional therapy that is commonly used in dermatology. While its systemic side effects, particularly those related to hepatic and lipid metabolism, are extensively documented, its impact on cardiac electrophysiology is little understood. Metabolic abnormalities are frequent, with hypertriglyceridemia occurring in up to 44% of patients, elevated total cholesterol in 30%, and transient liver enzyme elevation in approximately 10%–11%. This focused review summarizes the existing research on electrocardiographic (ECG) alterations associated with isotretinoin medication. Reported cardiac abnormalities are rare, with isolated case reports and small studies describing transient arrhythmias in fewer than 1%–2% of users, including premature ventricular contractions (PVCs), atrial tachycardia, and, in exceptionally rare instances, cardiomyopathy < 0.01%. Mechanistic ideas suggest changes in human ether-a-go-go-related gene (hERG) potassium channels, dyslipidemia-induced atherosclerosis, electrolyte imbalances, and sympathetic overactivity. However, large population-based studies have not found a significant increase in major cardiovascular events. Most ECG abnormalities, when noted, appear to be reversible with discontinuing isotretinoin. This review is unique in that it focuses on isotretinoin from both dermatological and cardiac viewpoints. It identifies important shortcomings in standardized ECG monitoring procedures and underscores the scarcity of large, prospective, controlled trials. Future research should focus on longitudinal, multicenter trials using serial ECG evaluations to better define risk profiles and guide clinical monitoring. Understanding infrequent but potentially serious cardiac effects is critical for improving isotretinoin's safety profile and guiding evidence-based therapy.

## Introduction

Acne vulgaris is a chronic inflammatory condition of the sebaceous unit that affects more than 80% of teenagers, with moderate-to-severe forms present in approximately 15%–20% of adolescents and young adults worldwide.^1^ Isotretinoin, a vitamin A derivative known as 13-cis-retinoic acid, is regarded as the gold-standard treatment for severe, refractory acne, and is prescribed worldwide to approximately 15%–25% of patients with moderate-to-severe disease and is currently the most effective acne treatment available.^2^ It addresses all four acne-related processes, including normalizing follicular desquamation, reducing sebaceous gland activity, inhibiting Propionibacterium acnes multiplication, and providing anti-inflammatory benefits.^3^ Although the hepatic and metabolic effects of isotretinoin, including hypertriglyceridemia (seen in up to 44% of patients) and increased cholesterol (30%), are widely documented, its cardiovascular consequences are less thoroughly investigated.^4^ Case studies detail cardiac occurrences, including sinus tachycardia, atrial tachycardia, premature ventricular contractions, and, infrequently, cardiomyopathy. Premature ventricular contractions (PVCs) are distinguished by an early beat, no preceding P wave, and a broad, aberrant QRS complex.^5^ Nonetheless, there are no standardized methods for cardiovascular monitoring, unlike the established standards for liver function and lipid assessment. This disparity is significant considering that isotretinoin is predominantly recommended to teens and young adults, a population for which long-term cardiovascular safety is especially pertinent.^4^

The goal of this focused review is to thoroughly evaluate and synthesize the corpus of literature on electrocardiogram (ECG) abnormalities in individuals receiving isotretinoin for severe acne, clarify the prevalence and clinical importance of documented events, and highlight the necessity for standardized cardiac monitoring methods throughout treatment.^2^, ^4^ While isotretinoin's systemic effects are widely understood and regularly observed, less is known about how it impacts cardiac function, particularly abnormal ECG readings. Given the significance of cardiovascular safety in long-term therapy, a targeted review is required to assemble new evidence, assess potential dangers, and guide clinical judgment. This approach ensures that the substantial but less obvious consequences are taken into account in overall safety assessments.

### Pharmacological profile of isotretinoin

Isotretinoin, a vitamin A derivative, is most commonly administered to treat severe, resistant acne. It targets and improves all of acne's major pathophysiological variables, such as inflammation, follicular hyperkeratinization, sebaceous gland hyperactivity, and Propionibacterium acnes colonization.^6^

Isotretinoin induces apoptosis in sebocytes, which is enhanced by the apoptotic proteins tumor necrosis factor-related apoptosis inducing ligand (TRAIL) and neutrophil gelatinase-associated lipoprotein. Reducing pro-inflammatory cytokines such as TNF-α, IL-4, IL-17, and interferon-γ helps reduce acne inflammation. It also alters the immune response by decreasing Th17 development while increasing regulatory T-cell activity.^7^, ^8^

Isotretinoin is a lipophilic compound with a large distribution volume in adipose tissue. 99.9% of the drug binds to plasma proteins, primarily albumin. When taken with a high-fat meal, its bioavailability improves. The cytochrome P450 enzymes CYP2C8, CYP2C9, CYP3A4, and CYP2B6 are primarily responsible for the drug's hepatic metabolism, which results in both active and inactive metabolites such as 4-oxo-isotretinoin, retinoic acid, and 4-oxo-retinoic acid. Urine and feces are the pathways by which these metabolites are conjugated and removed.^9^

Isotretinoin is associated with a number of negative side effects. In one study, 94% of participants identified cheilitis as the most common side effect. In terms of metabolism, it usually reduces high-density lipoprotein (HDL) while raising total cholesterol, low-density lipoprotein (LDL), and serum triglycerides.^4^, ^10^

Up to 11% of patients develop transient elevations in liver transaminases due to hepatic effects. Even though these increases are often moderate and self-limiting, they should be observed if they persist or worsen, as they may suggest underlying liver pathology.^9^

Although less frequently documented, cardiovascular adverse effects can have a significant clinical impact. Cases of sinus tachycardia have been documented to include atrial tachycardia, pericardial effusion, and temporary right bundle branch block. These findings emphasize the importance of cardiac surveillance in patients with pre-existing heart problems, despite their rarity.^5^ Please refer to Table 1 3, 4, 5, 6, 7, 8 for a summarized profile of isotretinoin.

**Table 1:** Metabolism and adverse effects of isotretinoin.^3^, ^4^, ^5^, ^6^, ^7^, ^8^

Category	Details
**Metabolism**	• Lipophilic, distributed in adipose tissue
	• ∼99.9% bound to plasma proteins (mainly albumin)
	• Bioavailability increases with high-fat meals
	• Metabolized in the liver by CYP2C8, CYP2C9, CYP3A4, CYP2B6
	• Active and inactive metabolites: 4-oxo-isotretinoin, retinoic acid, 4-oxo-retinoic acid
	• Excretion: urine and feces after conjugation
**Most common adverse effects**	• Cheilitis (up to 94%)
	• Hypertriglyceridemia (∼44%)
	• Increased total cholesterol (∼30%)
	• ↑ LDL, ↓ HDL
	• Transient liver transaminase elevations (∼11%)
**Less reported / Rare adverse effects**	• Cardiac: sinus tachycardia, atrial tachycardia, premature ventricular contractions (PVCs), right bundle branch block, pericardial effusion, dilated cardiomyopathy, renal infarction (very rare)
	• Electrolytes: hypokalemia, ↑ phosphorus, ↑ magnesium, ↑ zinc
	• Autonomic changes: transient ↑ sympathetic activity (SSR findings)

CYP, Cytochrome P450; HDL, high-density lipoprotein; LDL, low-density lipoprotein; PVC, premature ventricular contraction; SSR, sympathetic skin response.

### Cardiovascular implications of isotretinoin

Metabolically, isotretinoin therapy has been shown to significantly increase total cholesterol and triglyceride levels, which are established risk factors for atherosclerosis and cardiovascular disease.^11^ A study reported significant elevations in these lipid parameters during isotretinoin treatment, although no significant changes were observed in plasma phospholipids or red cell membrane fatty acid composition.^12^, ^13^

Direct cardiac implications have been observed in isolated cases.^14^ For instance, a case report detailed a 16-year-old boy who developed atrial tachycardia during isotretinoin therapy, with symptoms resolving upon discontinuation of the drug. Another study documented a 33-year-old woman experiencing premature ventricular contractions (PVCs) associated with isotretinoin use, which subsided after stopping the medication. Furthermore, a rare but severe case involved an 18-year-old male developing dilated cardiomyopathy and renal infarction after five months of isotretinoin therapy, with no other identifiable causes.^5^, ^14^, ^15^

Regarding the autonomic nervous system, isotretinoin may influence sympathetic activity. A study assessing sympathetic skin response (SSR) and electrocardiographic (ECG) parameters in patients undergoing isotretinoin treatment found increased sympathetic activity after one month of therapy, as indicated by changes in SSR, though no significant cardiac side effects were observed in ECG findings. This suggests that isotretinoin may transiently enhance sympathetic nervous system activity without causing overt cardiac dysfunction.^13^

Despite these reports, larger-scale studies have not demonstrated a significant association between isotretinoin use and increased cardiovascular risk.^16^ A population-based retrospective cohort study involving over 12,000 adults found no significant increase in cardiovascular events, including myocardial infarction, heart failure, or arrhythmias, among isotretinoin users compared to non-users.^17^ Similarly, a case-crossover study analyzing data from over 30,000 isotretinoin users did not find a statistically significant association between the drug and cardiovascular, cerebrovascular, or thromboembolic events.^14^, ^16^ Please refer to Table 2 for a summarized overview of all the mentioned studies.

**Table 2:** Reported cardiovascular cases associated with isotretinoin therapy.

Case	Event	Dose	Time of Onset	Outcome	Reversibility
**16-year-old boy (Hasdemir** et al., **2005)**^15^	Atrial tachycardia	Not specified	During therapy (≈3-months)	Symptom resolution after discontinuation	Yes, reversible
					
**33-year-old woman (Alan et al., 2016)**^5^	Premature ventricular contractions (PVCs)	Not specified	∼1-month into therapy	Arrhythmia resolved after drug withdrawal	Yes, reversible
					
**18-year-old male (Pepe et al., 2023)**^14^	Dilated cardiomyopathy + renal infarction + non-sustained VT	Not specified	After 5-months of isotretinoin	Severe cardiac dysfunction requiring discontinuation	Not fully reversible (serious outcome)
					
**Pediatric case (Kiliç et al., 2009)**^27^	Premature ventricular contractions (PVCs)	Not specified	During isotretinoin treatment	Resolved after discontinuation	Yes, reversible
					
**General reports (multiple cases, summarized)**	Sinus tachycardia, right bundle branch block, pericardial effusion	Variable	During treatment	Generally transient and non-lethal	Typically, reversible

PVC, Premature Ventricular Contraction; RBBB, Right Bundle Branch Block; VT, Ventricular Tachycardia.

### Overview of cardiac electrophysiology and ECG parameters

The possible effects of isotretinoin on cardiac electrophysiology have been meticulously researched, especially with regard to ECG measures like the QT interval, heart rate variability (HRV), T-wave abnormalities, and the risk of bradyarrhythmias in pediatric patients.

The time between the beginning of ventricular depolarization and the conclusion of repolarization is represented by the QT interval on an ECG. Clinically significant, prolonged corrected QT interval (QTc) can put people at risk for potentially fatal arrhythmias such as torsades de pointes. Adults normally have QTc values of less than 440 ms; values greater than 450 ms for males and 470 ms for females are regarded as prolonged.^18^

A study by Dursun et al. investigated the effects of isotretinoin on QT intervals and QT dispersion in 45 patients with severe acne undergoing six months of therapy at a dose of 0.8 mg/kg/day. The results demonstrated no significant changes in QT intervals or QT dispersion throughout the treatment period, suggesting that isotretinoin does not adversely affect these ECG parameters.^19^

Regarding HRV, which reflects autonomic nervous system activity and is an indicator of cardiac health, a study assessing sympathetic skin response (SSR) and ECG parameters in patients undergoing isotretinoin treatment found increased sympathetic activity after one month of therapy, as indicated by changes in SSR. However, no significant cardiac side effects were observed in ECG findings, indicating that while isotretinoin may transiently enhance sympathetic nervous system activity, it does not cause overt cardiac dysfunction. T-wave abnormalities, which can indicate repolarization disturbances, have not been consistently associated with isotretinoin therapy. While isolated case reports have documented arrhythmic events such as premature ventricular contractions (PVCs) and atrial tachycardia during isotretinoin use, these instances are rare and often resolve upon discontinuation of the drug.^5^

Therefore, current research indicates that isotretinoin medication does not significantly influence important ECG measures such as the QT interval, heart rate variability (HRV), or T-wave morphology in young patients, despite the fact that it may have an effect on some elements of autonomic function. Although large cohort studies generally report no significant ECG changes or increase in major cardiovascular events with isotretinoin, isolated case reports and small observational studies have documented arrhythmias and autonomic alterations. This discrepancy highlights the importance of vigilance, particularly in younger patients who may be more susceptible to drug-induced electrophysiological changes.

Kindly refer to Table 3 for a tabulated summary of ECG parameters in patients undergoing isotretinoin therapy.

**Table 3:** Summary of ECG parameters in patients undergoing isotretinoin therapy.

ECG Parameter	Clinical Relevance	Findings in Isotretinoin Studies	Implication
**QT Interval / QTc**	Prolongation can predispose to torsades de pointes and sudden cardiac death	No significant changes in QT interval or QT dispersion over 6 months of isotretinoin therapy.	Suggests isotretinoin does not prolong QT interval in most patients
			
**Heart Rate Variability (HRV)**	Marker of autonomic nervous system balance; reduced HRV indicates sympathetic dominance	SSR changes suggest transient increase in sympathetic activity; no ECG evidence of HRV changes	Possible transient autonomic modulation without clinically significant impact
			
**T-Wave Morphology**	Abnormalities may indicate repolarization issues and risk for arrhythmia	No consistent evidence of T-wave abnormalities in larger studies; isolated reports of arrhythmias exist	No established correlation with T-wave changes
			
**Bradyarrhythmias**	Slowed heart rhythm; can be drug-induced or related to conduction issues	No documented cases of isotretinoin-induced bradyarrhythmia	Not a common concern
			
**Premature Ventricular Contractions (PVCs)**	Can be benign or a precursor to serious arrhythmia depending on frequency and patient context	Documented in case reports; generally resolved after discontinuation of isotretinoin	Rare
			Reversible, may indicate individual susceptibility

HRV, Heart Rate Variability; PVC, Premature Ventricular Contraction; QTc, Corrected QT interval; SSR, Sympathetic Skin Response.

### Potential mechanisms behind ECG changes

**Figure 1:**
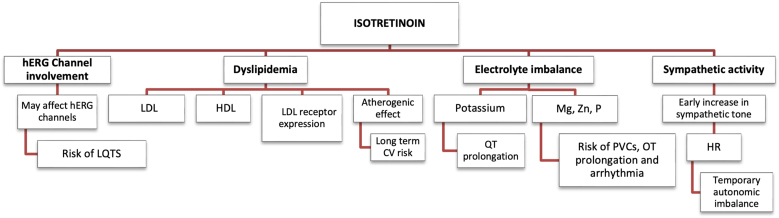
Influence of Isotretinoin on cardiac electrophysiology and ECG parameters. CV, Cardiovascular; ECG, Electrocardiogram; HDL, High-density lipoprotein; HR, Heart rate; HRV, Heart rate variability; Herg, human ether-à-go-go-related Gene; K, Potassium; LDL, Low-density lipoprotein; LQTS, Long QT syndrome; Mg, Magnesium; P, Phosphorus; PVCs, Premature ventricular contractions; Zn, Zinc.

Isotretinoin has been linked to slight but important cardiac electrophysiological changes. To understand these effects, the existing evidence can be separated into two categories: preclinical (experimental) data and human clinical findings. Preclinical research, primarily using embryonic or cellular models, suggests that probable mechanisms include direct impacts on cardiac ion channels, particularly human hERG potassium channels, and interference with myocardial development. Human clinical evidence, on the other hand, emphasizes metabolic and autonomic pathways such as dyslipidemia, electrolyte imbalances, and transitory sympathetic overactivity, all of which may have an indirect effect on cardiac conduction and ECG parameters. Although arrhythmias are rare, studies have explored different biological reasons that might explain changes seen on ECGs during treatment, as seen in Fig. 1.

#### Experimental/Preclinical evidence

The hERG potassium channels have a role in heart repolarization. These channels serve to avoid arrhythmias by gradual deactivation and quick voltage-dependent inactivation. The majority of cases of acquired LQTS result from hERG inhibition.^20^ Although isotretinoin has not been linked to hERG blocking, its systemic pharmacokinetics and class structure raise worries that it may influence these channels accidentally. More research is needed to investigate the potential interaction between isotretinoin and hERG channels, as numerous non-cardiac drugs have been linked to LQTS via their effects on hERG channels.

Research on chick embryos demonstrated that isotretinoin exposure delayed myocardial contractions and slowed the growth of cardiac cells. This suggests that the drug could directly affect heart development even though these findings are from embryos.^13^, ^21^

#### Human clinical data

Prospective cohort studies have shown that isotretinoin causes dyslipidemia, increasing LDL and decreasing HDL levels. Further evidence provided by Karapinar et al., demonstrated that isotretinoin upregulated both LDL levels and LDL receptor expression.^22^, ^23^ The study found that after three months of treatment with isotretinoin, potassium levels decreased and phosphorus, magnesium, and zinc levels increased. Hypokalemia, in particular, is known to put people at risk for cardiac arrhythmias, like QT prolongation and premature ventricular contractions.^22^ Additionally, during the early stages of treatment, isotretinoin may increase the activity of the sympathetic nervous system and affect the autonomic balance. This can influence cardiac electrophysiology and be the cause of temporary flare-ups of acne. Evidence of this modulation has been provided through sympathetic skin response analysis, revealing a rise in sympathetic activity during the first month of treatment.^24^

Overall, experimental studies suggest that isotretinoin may affect cardiac electrophysiology via mechanisms including hERG channel interaction and impaired myocardial development, whereas clinical data point to dyslipidemia, electrolyte imbalances, and transient autonomic changes. However, the evidence is fragmented, with animal studies lacking direct clinical correlation and human studies limited to small cohorts or isolated case reports. There is a lack of large-scale, prospective trials with standardized ECG monitoring, and no definitive guidelines are available for cardiovascular surveillance. These gaps highlight that, in order to determine if isotretinoin presents a significant risk of arrhythmia in clinical practice, systematic research is required.

### Risk stratification and clinical relevance

More frequent laboratory monitoring may be necessary for patients with higher body weight, as studies have shown that these individuals are at increased risk of developing laboratory abnormalities during isotretinoin therapy, including elevated lipid levels and liver enzyme disturbances.^25^ This increased susceptibility may be due to differences in drug distribution, metabolism, or underlying metabolic conditions. In contrast, current evidence supports a more tailored approach for otherwise healthy individuals with normal baseline investigations, for whom less frequent laboratory monitoring appears to be both safe and cost-effective.^25^ Such a stratified approach to monitoring may improve patient compliance and reduce unnecessary healthcare costs without compromising safety.

**Figure 2:**
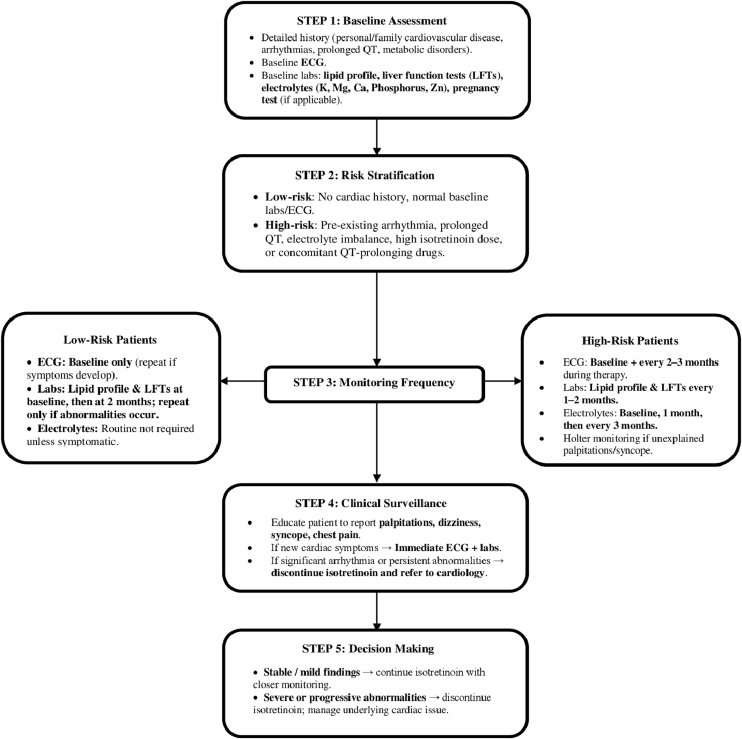
Clinical management algorithm for isotretinoin in patients at cardiovascular risk. Ca, Calcium; ECG, Electrocardiogram; K, Potassium; LFTs, Liver function tests; Mg, Magnesium; QT, QT Interval; Zn, Zinc.

In addition to systemic effects, emerging case reports have highlighted potential cardiovascular adverse events associated with isotretinoin, including PVCs and atrial tachycardia.^15^ These arrhythmias developed during the course of treatment and were not present prior to isotretinoin initiation. Importantly, in both cases, the symptoms resolved completely following discontinuation of the drug, suggesting a reversible and potentially drug-induced mechanism. The temporal relationship between the onset of these cardiac symptoms and isotretinoin use strengthens the hypothesis of a causal association. Although causality cannot be definitively established from isolated reports, the consistency in clinical course and resolution upon drug withdrawal indicates that isotretinoin may exert arrhythmogenic effects in susceptible individuals. These findings underscore the need for greater awareness of potential cardiac risks, especially in patients with a personal or family history of arrhythmias, and may warrant further investigation into routine cardiac screening protocols during isotretinoin therapy.^15^ Please refer to Fig. 2 for an algorithm of the clinical management for patients using isotretinoin at cardiovascular risk.

Because so few patients experience markedly aberrant reactions to isotretinoin medication, routine blood testing during treatment is not required. It has been proposed in some studies that most patients should only undergo restricted blood testing (includes hepatic function test, lipid profile and pregnancy test), whereas those who exhibit a notable change in their liver enzymes, cholesterol, or triglycerides should undergo more thorough blood testing.^26^ Refer to Table 4 for a detailed overview of the risk factors associated with ECG changes during isotretinoin therapy, and Table 5 for recommendations for clinical practice and monitoring.

**Table 4:** Risk factors for ECG changes during isotretinoin use.

Risk Factor	Description	Clinical Consideration / Monitoring
**High Isotretinoin Dose**	Daily dose ≥ 1 mg/kg/day, or cumulative dose > 120–150 mg/kg (upper end of standard range). Higher plasma levels are associated with more frequent lab abnormalities and theoretical cardiac risk.	• Baseline ECG and labs (lipids, LFTs, electrolytes).
		• Repeat ECG at 1–3 months if high-dose regimen.
		• Monthly lipid and LFT monitoring for first 2–3 months, then q2–3 months.
**Pre-existing Cardiac Conditions**	History of arrhythmia, congenital conduction defects, cardiomyopathy, structural heart disease.	• Baseline cardiology consult and ECG.
		• Repeat ECG at 1 and 3 months, then q3–6 months.
		• Closer follow-up if symptomatic.
**Electrolyte Imbalance**	Hypokalemia, hypomagnesemia, hypocalcemia, or trace element disturbances (Zn, P). These may potentiate arrhythmias.	• Correct electrolyte disturbances before starting.
		• Monitor electrolytes at baseline and periodically (every 2–3 months, or sooner if symptomatic).
**Concomitant Medications**	Especially QT-prolonging drugs (e.g., macrolides, fluoroquinolones, antipsychotics, antiarrhythmics).	• Review drug history.
		• Avoid combining with other QT-prolonging agents when possible.
		• ECG monitoring at baseline and if new QT-prolonging drug is added.
**Age and Autonomic Tone**	Younger patients may have higher vagal tone and autonomic variability; adolescents/young adults form the bulk of isotretinoin users.	• Baseline ECG.
		• Educate patients on palpitations, syncope, chest discomfort.
		• No additional routine ECG unless symptomatic or other risk factors present.

ECG, Electrocardiogram; HRV, Heart rate variability; QT, QT interval.

**Table 5:** Recommendations for clinical practice and monitoring.

Recommendation	Rationale	Implementation
**Baseline ECG before starting isotretinoin**	To detect pre-existing abnormalities	Routine screening
**Monitor HR and QTc in high-risk patients**	Identify clinically significant changes	Periodic ECG checks
**Electrolyte panel monitoring**	Avoid confounding cardiac effects	Especially if symptomatic
**Patient education on cardiac symptoms**	Prompt detection of palpitations/syncope	During consent/counseling

ECG, Electrocardiogram; HR, Heart rate; QTc, Corrected QT interval.

### Comparison with other systemic acne treatments

In addition to isotretinoin, systemic antibiotics, hormone therapies, and other retinoids are commonly used to treat acne. Tetracyclines, such as doxycycline and minocycline, are the principal oral antibiotics. As stated by Dreno et al., “The preferred treatment for acne involves tetracycline antibiotics because of their potent anti-inflammatory properties, such as inhibiting neutrophil chemotaxis and lowering proinflammatory cytokine production”.^2^ Although commonly used, they are generally not linked to ECG irregularities.

Hormonal therapies, such as Combined Oral Contraceptives (COCs) and spironolactone, are especially effective for women with hormonal acne and can be used alongside antibiotics.^2^ Although these agents affect vascular tone and coagulation, they have not demonstrated reliable ECG effects in otherwise healthy people.^27^

Isotretinoin, on the other hand, remains the most comprehensive treatment, targeting all four basic acne processes. It is the only medication acknowledged for targeting the four pathogenic mechanisms of acne.^2^ In a six-month study, all patients maintained sinus rhythm with no atrial arrhythmias. Another study observed no significant difference in ECG parameters before and after therapy.^28^ Nonetheless, rare cardiac events, such as premature ventricular contractions, atrial tachycardia, and dilated cardiomyopathy, have been documented.^29^

Isotretinoin distinctly lowers sebum production and influences sebocyte activity at the molecular level, unlike other retinoids such as adapalene or tazarotene. This differentiates it from the typical class effects observed in retinoid therapy, providing a more lasting and thorough strategy for managing acne.^30^ Please refer to Table 6 for the comparative adverse effect profiles and cardiovascular safety of the main systemic acne treatments.

**Table 6:** Comparative adverse effect profiles and cardiovascular safety of main systemic acne treatments.

Drug/Class	Common non-cardiac adverse effects	Cardiac/Cardiovascular adverse events	Risk Magnitude & Reversibility
**Isotretinoin**	Cheilitis (≈94%), xerosis, dry eyes, arthralgia, transient ↑LFTs, ↑TG/LDL, ↓HDL	Rare: atrial tachycardia, PVCs, transient RBBB, pericardial effusion, dilated cardiomyopathy (isolated). Dyslipidemia may increase atherosclerotic risk long-term.	Population studies: no increase in major CV events. Most arrhythmias reversible after discontinuation.
**Tetracyclines (doxycycline, minocycline)**	GI upset, photosensitivity (doxy), vestibular symptoms (mino), hypersensitivity/ DRESS (mino)	Generally, QT-neutral (doxycycline not QT-prolonging). Very rare: minocycline-induced eosinophilic myocarditis within hypersensitivity syndromes.	No population-level signal for CV events. Myocarditis rare, improves after cessation + immunomodulation.
**Combined Oral Contraceptives (COCs)**	Nausea, breast tenderness, breakthrough bleeding, headache	Venous thromboembolism (VTE): 2–3× baseline risk. ↑ risk of arterial events (MI, stroke) in smokers > 35, or with migraine with aura. No QT/ECG effect.	Absolute VTE risk: ∼7–10/10,000 women-years vs. 4/10,000 non-users. Risk falls rapidly after discontinuation.
**Spironolactone (female acne)**	Menstrual irregularities, breast tenderness, fatigue, diuresis	Hyperkalemia (rare in healthy young women; higher in CKD/with RAAS blockers). No intrinsic proarrhythmic signal; some evidence of QTc shortening in cardiac cohorts.	Low risk in healthy women. Lab abnormalities usually within weeks, reversible with dose adjustment or cessation.

CKD, Chronic kidney disease; COCs, Combined oral contraceptives; CV, Cardiovascular; DRESS, Drug reaction with eosinophilia and systemic symptoms; ECG, Electrocardiogram; GI, Gastrointestinal; HDL, High-density lipoprotein; LDL, Low-density lipoprotein; LFTs, Liver function tests; MI, Myocardial infarction; PVCs, Premature ventricular contractions; QTc, Corrected QT interval; RAAS, Renin-angiotensin-aldosterone system; RBBB, Right bundle branch block; TG, Triglycerides; VTE, Venous thromboembolism.

### Limitations of current evidence

The existing research examining ECG changes in patients on isotretinoin treatment is limited by various methodological flaws. A significant amount of the accessible data comes from individual case reports and limited observational studies, which diminishes the external validity and strength of their findings. These reports frequently detail rare cardiac symptoms, like atrial tachycardia, QT interval elongation, or premature ventricular contractions, without utilizing standardized monitoring or comparison control groups.^27^

Furthermore, there is a substantial lack of planned clinical trials that incorporate systematic ECG measurements throughout treatment. Many current studies use before-and-after designs with small sample sizes, limiting the capacity to draw statistically meaningful conclusions about arrhythmic risk. Differences in dosage, treatment duration, and subject characteristics among trials make interpretation more difficult.^31^

However, future clinical trials assessing ECG abnormalities associated with oral isotretinoin should use standardized monitoring and design criteria to improve methodological rigor. Trials should include a baseline ECG and monthly ECG assessments throughout the treatment period to detect early electrophysiological changes similar to the temporal pattern seen in laboratory abnormalities such as triglyceride and transaminase elevations, which appear within the first 1‒3 months of therapy. A follow-up ECG (≥1-month after cessation) is recommended to assess reversibility. Uniform laboratory monitoring, focusing on lipids at baseline and months 1‒2, as well as hepatic and triglyceride markers at baseline, and as clinically prescribed, would be needed as a part of an efficient monitoring protocol. Importantly, the inclusion of control groups, whether placebo or active comparators (e.g., doxycycline, spironolactone, or COCs), is required for allocating observed ECG alterations to isotretinoin. Trial participants should be followed for at least 3‒6 months after therapy to detect delayed arrhythmias or conduction abnormalities. Core outcomes should include the frequency and severity of QTc prolongation, arrhythmias, and conduction anomalies, as well as any symptom-related observations. This systematic method would considerably improve the evidence base, allowing the discovery of rare or delayed occurrences, and allow for rigorous comparisons of therapies.^32^, ^33^, ^34^

A significant concern is the possibility of publication bias. Reports and studies detailing unusual or new cardiovascular effects are more prone to publication, while results showing no heart abnormalities might not be published. This biased reporting may result in an inflated perception of isotretinoin-related ECG irregularities in the published studies.^31^

## Conclusion

Current research on the association between isotretinoin use and ECG abnormalities is insufficient. While isolated case reports and small observational studies have revealed possible arrhythmic effects, larger cohort studies and systematic reviews have not found a consistent rise in significant adverse cardiovascular events. These disparities are most likely due to methodological limitations such as limited sample sizes, varied demographics, short follow-up periods, and a lack of uniform ECG monitoring. As a result, there is currently no definitive causal relationship between isotretinoin and clinically severe arrhythmias. Until stronger evidence emerges, doctors should prescribe isotretinoin cautiously and evidence-based. A baseline cardiovascular evaluation, including a history and an ECG, is recommended for patients with known arrhythmias, prolonged QTc, electrolyte problems, or concurrent use of QT-prolonging medicines. Laboratory monitoring should adhere to consensus standards, with lipid profile and liver function tests performed at baseline and repeated every one to two months, while further testing might be customized based on anomalies or dose escalation. In low-risk patients, routine repeat ECGs are unnecessary; however, in high-risk individuals, periodic monitoring every two to three months may be indicated. Patients should also be advised to report any palpitations, dizziness, syncope, or chest discomfort as soon as possible, as these should prompt an ECG and laboratory evaluation. If new, clinically significant arrhythmias or ECG abnormalities develop during therapy, discontinue isotretinoin and refer the patient to cardiology for further evaluation. In conclusion, despite isolated cardiac abnormalities, isotretinoin has a very positive risk-benefit profile. It remains the only medication capable of addressing all four primary pathogenic mechanisms of acne, including follicular hyperkeratinization, excess sebum production, inflammation, and *Cutibacterium acnes* growth, providing unrivaled and frequently permanent remission in severe and refractory acne. The recommended monitoring measures are therefore not intended to limit its usage, but rather to improve patient safety and maintain confidence in a medicine with unsurpassed therapeutic efficacy in dermatology. By incorporating selective cardiovascular screening into clinical practice, clinicians can continue to reap the transformative benefits of isotretinoin while being cautious for uncommon but tolerable cardiac side effects.

## ORCID ID

Afra Wasama Islam: 0009-0007-4855-6226

Prarthana Motdhare: 0009-0007-6061-3537

Shreya Mohan: 0009-0007-1452-332X

Swapna Bejoy: 0009-0008-4652-4148

## Research data availability

The entire dataset supporting the results of this study was published in this article.

## Financial support

None declared.

## Authors' contributions

Afra Wasama Islam: Literature search; writing original draft; editing; conceptualization; approval of the final version of the manuscript.

Prarthana Motdhare: Literature search; writing original draft; editing; approval of the final version of the manuscript.

Shreya Mohan: Literature search; writing original draft; editing; approval of the final version of the manuscript.

Swapna Bejoy: Literature search; writing original draft; editing; approval of the final version of the manuscript.

Harsahaj Singh Wilkhoo: Editing; literature search; writing; supervision; correspondence; proofreading; approval of the final version of the manuscript.

## Conflicts of interest

None declared.

## References

[1] Costa C.S., Bagatin E., Martimbianco A.L.C., da Silva E.M., Lúcio M.M., Magin P. (2018). Oral isotretinoin for acne. Cochrane Database Syst Rev..

[2] Zaenglein A.L., Pathy A.L., Schlosser B.J., Alikhan A., Baldwin H.E., Berson D.S. (2016). Guidelines of care for the management of acne vulgaris. J Am Acad Dermatol..

[3] Li C., Chen J., Wang W., Ai M., Zhang Q., Kuang L. (2019). Use of isotretinoin and risk of depression in patients with acne: a systematic review and meta-analysis. BMJ Open..

[4] Brito M. de F. de M., Sant’Anna I.P., Galindo J.C.S., Rosendo L.H.P. de M., Santos J.B. dos (2010). Evaluation of clinical adverse effects and laboratory alterations in patients with acne vulgaris treated with oral isotretinoin. An Bras Dermatol..

[5] Alan S., Ünal B., Yildirim A. (2016). Premature ventricular contractions associated with isotretinoin use. An Bras Dermatol..

[6] Vasam M., Korutla S., Bohara R.A. (2023). Acne vulgaris: a review of the pathophysiology, treatment, and recent nanotechnology-based advances. Biochem Biophys Rep..

[7] Aoki K.C., Smithy W., Au S., Bartos S. (2024). Recalcitrant pustular dermatosis successfully treated with prolonged isotretinoin therapy. Cureus..

[8] Melnik B.C. (2017). Apoptosis may explain the pharmacological mode of action and adverse effects of isotretinoin, including teratogenicity. Acta Derm Venereol..

[9] Tawanwongsri W., Kanchanasuwan T., Eden C. (2025). Isotretinoin and hepatotoxicity in patients with acne: a narrative review. Cosmetics..

[10] Ahmadv H., Javanbakht A.M.A., Pour H.M. (2011). Effects of oral isotretinoin on serum lipids and gamma glutamyl transpeptidase activity in acne vulgaris patients. AJPP..

[11] Ostlere L.S., Harris D., Morse-Fisher N., Wright S. (1996). Effect of systemic administration of isotretinoin on blood lipids and fatty acids in acne patients. Int J Dermatol..

[12] Karadag A.S., Gumrukcuoglu H.A., Gunes Bilgili S., Ozkol H.U., Ertugrul D.T., Simsek H. (2012). Does isotretinoin therapy have any effects on electrocardiography, heart rate and blood pressure?. J Dermatolog Treat..

[13] Ay H., Aksoy M., Güngören F. (2019). Assessment of autonomic nervous system functions and cardiac rhythms in patients using isotretinoin. Postepy Dermatol Alergol..

[14] Pepe M., Napoli G., Carella M.C., De Feo D., Tritto R., Guaricci A.I. (2023). A young patient presenting with dilated cardiomyopathy and renal infarction during treatment with isotretinoin: mere coincidence or serious side effect of a drug commonly used in adolescence?. Diagnostics (Basel)..

[15] Hasdemir C., Sagcan A., Sekuri C., Ildizli M., Ulucan C., Ceylan C. (2005). Isotretinoin (13-cis-retinoic acid) associated atrial tachycardia. Pacing Clin Electrophysiol..

[16] Bérard A., Azoulay L., Nakhai-Pour H.R., Moussally K. (2011). Isotretinoin and the risk of cardiovascular, cerebrovascular and thromboembolic disorders. Dermatology..

[17] Ghanshani S., Chen C., Lin B., Zhou H., Lee M.S. (2021). Isotretinoin and risk of cardiovascular events in adults with acne: a population-based retrospective cohort study. Am J Clin Dermatol..

[18] Baumert M., Porta A., Vos M.A., Malik M., Couderc J.P., Laguna P. (2016). QT interval variability in body surface ECG: measurement, physiological basis, and clinical value: position statement and consensus guidance endorsed by the european heart rhythm association jointly with the ESC working group on cardiac cellular electrophysiology. Europace..

[19] Dursun R., Alpaslan M., Caliskan M., Ciftci O., Kulaksizoglu S., Seckin D. (2011). Isotretinoin does not prolong QT intervals and QT dispersion in patients with severe acne: a surprising finding for a drug with numerous side effects. J Drugs Dermatol..

[20] Perry M.D., Ng C.A., Mann S.A., Sadrieh A., Imtiaz M., Hill A.P. (2015). Getting to the heart of hERG K(+) channel gating. J Physiol..

[21] Wiens D.J., Mann T.K., Fedderson D.E., Rathmell W.K., Franck B.H. (1992). Early heart development in the chick embryo: effects of isotretinoin on cell proliferation, alpha-actin synthesis, and development of contractions. Differentiation..

[22] Karapınar T., Polat M., Buğdaycı G. (2020). Evaluation of subclinical atherosclerosis in Turkish patients with acne vulgaris receiving systemic isotretinoin. Dermatol Ther..

[23] Cowan T.L., Stark M., Sarmiento S., Miller A. (2025). Systematic review of rare major adverse cardiovascular events associated with the treatment of acne with isotretinoin. Australas J Dermatol..

[24] Akman T.C., Yazici M., Atila A., Mertoglu C. (2024). Analysis of isotretinoin-induced alterations in the levels of plasma trace elements: investigation of the relationship between magnesium, phosphorus, potassium, zinc, and treatment-related side effects. Biol Trace Elem Res..

[25] Alajaji A., Alrawaf F.A., Alosayli S.I., Alqifari H.N., Alhabdan B.M., Alnasser M.A. (2021). Laboratory abnormalities in acne patients treated with oral isotretinoin: a retrospective epidemiological study. Cureus..

[26] Altman R.S., Altman L.J., Altman J.S. (2002). A proposed set of new guidelines for routine blood tests during isotretinoin therapy for acne vulgaris. Dermatology..

[27] Kiliç E., Sahin M., Sahin S., Ozer S. (2009). Isotretinoin (13-cis-retinoic acid)-associated premature ventricular contractions. Turk J Pediatr..

[28] Teo S.Y.M., Kanaley J.A., Guelfi K.J., Marston K.J., Fairchild T.J. (2020). The effect of exercise timing on glycemic control: a randomized clinical trial. Med Sci Sports Exerc..

[29] Chivot M. (2005). Retinoid therapy for acne. A comparative review. Am J Clin Dermatol..

[30] Bielli A., Scioli M.G., D’Amico F., Tarquini C., Agostinelli S., Costanza G. (2019). Cellular retinoic acid binding protein-II expression and its potential role in skin aging. Aging (Albany NY)..

[31] Ioannidis J.P.A. (2005). Why most published research findings are false. PLOS Med..

[32] Emtenani S., Abdelghaffar M., Ludwig R.J., Schmidt E., Kridin K. (2024). Risk and timing of isotretinoin-related laboratory disturbances: a population-based study. Int J Dermatol..

[33] Park Y.J., Shin H.Y., Choi W.K., Lee A.Y., Lee S.H., Hong J.S. (2023). Optimal laboratory testing protocol for patients with acne taking oral isotretinoin. World J Clin Cases..

[34] Xia E., Han J., Faletsky A., Baldwin H., Beleznay K., Bettoli V. (2022). Isotretinoin laboratory monitoring in acne treatment: a delphi consensus study. JAMA Dermatol..

